# Is the use of computer navigation in total knee arthroplasty improving implant positioning and function? A comparative study of 198 knees operated at a Norwegian district hospital

**DOI:** 10.1186/1471-2474-14-321

**Published:** 2013-11-14

**Authors:** Gro Sævik Dyrhovden, Øystein Gøthesen, Stein Håkon Låstad Lygre, Anne Marie Fenstad, Tor Egil Sørås, Svein Halvorsen, Truls Jellestad, Ove Furnes

**Affiliations:** 1 The Norwegian Arthroplasty Register, Department of Orthopedic Surgery, Haukeland University Hospital, Bergen, Norway; 2 Department of Radiology, Haukeland University Hospital, Bergen, Norway; 3 Department of Orthopedic Surgery, Lærdal Hospital, Helse Førde HF, Lærdal, Norway; 4 Department of Orthopedic Surgery, Haugesund Hospital, Helse Fonna HF, Haugesund, Norway; 5 Departement of Clinical Medicine 2, Faculty of Medicine and Dentistry, University of Bergen, Bergen, Norway; 6 Department of Occupational Medicine, Haukeland University Hospital, Bergen, Norway

**Keywords:** Computer navigation, Total knee arthroplasty, KOOS, EQ-5D, Quality of life

## Abstract

**Background:**

There are few Scandinavian studies on the effect of computer assisted orthopedic surgery (CAOS) in total knee arthroplasty (TKA), compared to conventional technique (CON), and there is little information on effects in pain and function scores. This retrospective study has evaluated the effects of CAOS on radiological parameters and pain, function and quality of life after primary TKA.

**Methods:**

198 primary TKAs were operated by one surgeon in two district hospitals; 103 CAOS and 95 CON. The groups were evaluated based on 3 months post-operative radiographs and a questionnaire containing the knee osteoarthritis outcome score (KOOS), the EQ-5D index score and a visual analogue scale (VAS) two years after surgery. Multiple linear regression method was used to investigate possible impact from exposure (CON or CAOS).

**Results:**

On hip-knee-ankle radiographs, 20% of measurements were > ±3° of neutral in the CAOS group and 25% in the CON group (p = 0.37). For the femoral component, the number was 5% for CAOS and 18% for CON (p < 0.01). For the tibial component, the difference was not statistically significant (p = 0.58). In the sagittal plane, the surgeon tended to apply more femoral flexion and more posterior tibial slope with CAOS. We observed no statistically or clinically significant difference in KOOS score, VAS or ∆EQ-5D (all p values >0.05), but there was a trend towards better scores for CAOS. Operation time was 3 minutes longer for CON (p = 0.37).

**Conclusions:**

CAOS can improve radiological measurements in primary TKA, and makes it possible to adjust component placement to the patient’s anatomy. Over-all, the two methods are equal in pain, function and quality-of-life scores.

## Background

There is an ongoing discussion whether the use of computer assisted orthopedic surgery (CAOS) can improve the radiological or clinical results of total knee artroplasty (TKA).

Some studies have reported that CAOS improves the alignment of the components in TKA compared to conventional technique (CON) [1,2]. More than ±3° malalignment is reported to have a poorer outcome in function and survival [3,4]. A meta-analysis reported a reduction in rate of outliers (defined as more than 3° malalignment varus or valgus) when operated with CAOS of approximately 80% in limb mechanical axis (from 18.6% to 4.3%), and 87% (from 18.4% to 3.1%) and 80% (from 12.2% to 3.5%) for the femoral and tibial component, respectively [5]. On the other hand, an analysis on data from the Norwegian Arthroplasty Register (NAR) has shown a higher relative risk of revision for computer assisted TKA in a short-term follow-up of two years, compared to conventionally operated TKA [6].

Few papers have been published in Scandinavia, and there is also little information about the patients’ pain, function and quality of life using CAOS. The learning curve of CAOS has been an issue [7,8], and few studies have been published with one single surgeon, proficient in both methods.

The aim of this retrospective study was to assess the effects of CAOS on the radiological alignment of the components, and also pain- and function scores. The patients were operated in the same period, performed by one single surgeon, experienced in both techniques.

## Methods

The study population was based on 198 primary TKAs operated in the district hospitals in Lærdal and Førde; 103 CAOS and 95 CON. The two groups were operated during the same period; the patients in the CON group were operated between 2006/10/05 and 2008/08/27, and the CAOS group was operated between 2006/11/28 and 2008/12/30. All patients operated by the current surgeon in this period were included. In all CAOS procedures, the navigation system VectorVision Kolibri; BrainLab was used. CAOS was used in all patients when the computer was available to the surgeon. The patients in the CON group were partly operated before the computer was received in Lærdal. In order to get enough patients in the CON group, some patients were also included after introducing CAOS. These were operated when the computer was used by other surgeons or in another hospital. No specific inclusion- or exclusion criteria were used.

All patients were operated by the same surgeon, who had performed about 500 TKAs with CON and 700 with CAOS at the beginning of this study. The prosthesis Profix CR (Smith and Nephew) was used in all the TKAs, and the patients received equal post-operative treatment and rehabilitation. Both cemented and uncemented implants were included. Patella was not resurfaced in any operations. In the CON group, the femoral component was cut in 5 or 7 degrees valgus relative to the intramedullary rod. The cutting block was selected in order to maintain the patient’s original anatomy. For the tibial component, the posterior slope was cut at 4 degrees relative to the intramedullary rod.

**Figure 1:**
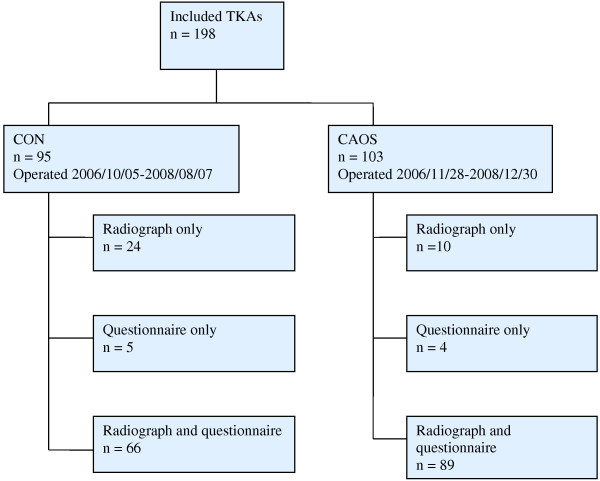
**Flow diagram of patients.** Overview of the number of patients, radiographs and questionnaire in each group. The patients were operated during the period 05.10.06 to 30.12.08. CON = conventional technique, CAOS = computer assisted orthopedic surgery.

Post-operative radiographs were taken within 3 months after surgery, according to the standard regimes at the hospital. In addition, we have evaluated the patients’ function, pain and quality of life in the two groups, based on self-administered questionnaires. An overview of the number of patients, radiographs and questionnaire in each group is presented in Figure 1.

The questionnaires were sent to the patients minimum two years post-operatively to ensure that the results of the intervention had stabilized [9]. Supplementing demographic information about the patient (diagnosis, age, sex, ASA-class, fixation and operation time) was collected from the NAR.

### Radiographs

**Figure 2:**
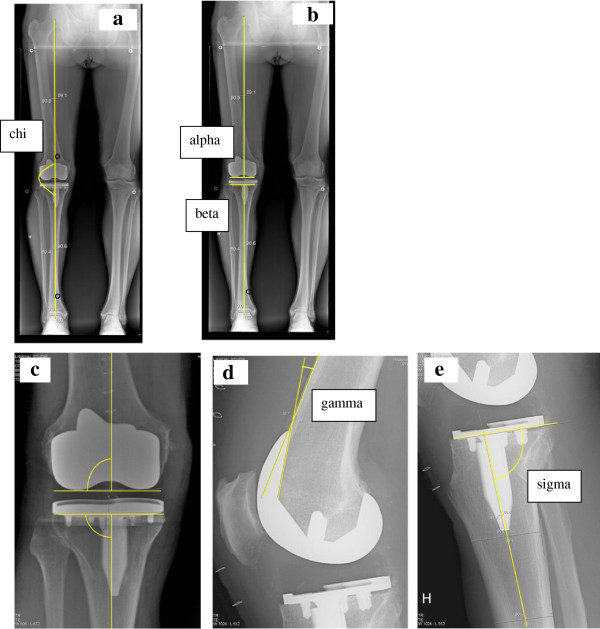
**Radiological measurements.** 2**a**: Drawing tools were used to mark the centre of the femoral head, the knee and talus. Lines connecting these centers define the mechanical axis (*chi*). The angle is measured on the lateral side. Angles <180° indicate valgus, >180° indicate varus. 2**b**: Overview of the *alpha* and *beta* angles, which measure the femoral and tibial components in the frontal plane. *Alpha* is measured between a line from the centre of the femoral head to the centre of distal femur and a line parallel to the femoral condyles. *Beta* is measured between a line from the centre of talus to the centre of proximal tibia and a line along the plateau of tibial component. 2**c**: The centre of distal femur is defined as the point where a line parallel to the femoral condyles crosses a perpendicular line from the centre of femoral notch. The centre of proximal tibia is defined as the centre of the plateau of the tibial component. 2**d**: The *gamma* angle is measured between the frontal femoral cortex and the inner frontal part of the femoral component. A large angle indicates high degree of femoral component flexion. 2**e**: The tibial slope is measured between the centre of tibia and the plateau of the tibial component, defined as the *sigma* angle. An angle <90° indicates posterior slope of the tibial component.

Radiological parameters were measured on postoperative hip-knee-ankle (HKA) radiographs in the frontal plane with the patient in standing position [10] and in the sagittal plane with flexed knee 10° to 20°, according to standard regimes of post-operative imaging at the hospital (Lærdal Hospital and Førde Hospital). The radiographs were sent on CDs to Haukeland University Hospital, and thereafter deidentified in the scientific server at the radiological department before measuring. The measurements were done according to the description in Figure 2.

In the frontal plane, the following angles were measured: the mechanical axis of the leg [11] (*chi*; Figure 2a) and the component alignment for the femoral (*alpha*; Figure 2b-c) and tibial (*beta*; Figure 2b-c) components [11,12]. In the sagittal plane, following angles were measured: the sagittal femoral component angle (*gamma*; Figure 2d) and the sagittal tibial component angle (*sigma*; Figure 2e) [13]. According to surgical plan the ideal value of *chi*, *alpha* and *beta* were 180, 90 and 90 degrees, respectively. In the CON group, the ideal *gamma* angle was 0-10°, whereas an ideal *sigma* angle was 86°. Sagittal alignments in the CAOS group were individually adjusted to the patient’s original anatomy, measured by the surgeon on preoperative radiographs.

The angles were measured by an independent observer. All angles in the frontal plane were measured on the lateral side. The measurements of the angles were determined by using drawing tools in Impax DS3000 (AGFA), and registered continuously in a database.

Radiographs were available on 189 of the 198 knees. Radiological parameters were measured on 90 knees in the CON group and 99 in the CAOS group, whereas radiographs on 9 knees were missing (Figure 1).

### Questionnaire

The questionnaire consisted of the validated Norwegian translation of the knee-specific knee injury and osteoarthritis outcome score (KOOS) (The translation can be found at http://www.koos.nu). The questionnaire also included questions considering general health factors, needed to calculate the Charnley category [14,15] applied to knee arthroplasty patients and the EQ-5D index score, which is a valid and reliable instrument for health quality measurement [16,17]. The EQ-5D was filled in twice, to get both pre-operative score and the score at time of investigation. The patients were also asked to fill in a Visual Analogue Scale (VAS) concerning “pain from the operated knee” the previous month, and a VAS to describe “satisfaction with the surgery”. The self-administrated questionnaire was sent to the patient in June 2010 with an information letter, and the patients willing to attend returned the questionnaire with a signed consent form (to participate in the study).

### Statistics

The primary outcome measures were the number of outliers (defined as more than ±3° from the ideal angle measurement) for each angle measurement, in addition to the KOOS scores, VAS and ∆EQ-5D.

Based on previous studies, we expected a larger divergence of the measured angles in the CON group (SD = 1.3) compared to the CAOS group (SD = 0.9) [2]. A power analysis concluded that we needed 79 patients in each group to achieve 80% power and a significance level of 0.05.

Minimal perceptible clinical difference is 8 to 10 points for KOOS subscales [18] and 9 to 12 units for a visual analogue scale [19]. For the KOOS subscales, a difference of 8 to 10 points is considered clinically relevant. Nine to 12 units is minimal perceptible change to patients with knee osteoarthritis [19]. To have an 80% chance of detecting as significant (at the two-sided 5% level) a ten-point difference in mean KOOS subscales [18], with an assumed standard deviation of 20 [20], 64 individuals in each treatment group were required. We also analyzed outcome in each of the 42 detailed questions from KOOS [21]. A difference of more than 0.4 points was considered clinically significant, whereas statistical significance level was set at 0.001 after performing a Bonferroni correction.

Differences in sex, Charnley category, fixation and diagnosis were analyzed with the Pearson chi-square test. To estimate differences in age, pre-operative EQ-5D, operation time and radiological parameters, student t-test was used. Pearson chi-square test was used to find differences in number of outliers. In the analyses, multiple linear regression method was used to investigate possible impact from exposure (CON or CAOS). These analyses were adjusted for possible confounding from age, sex, fixation, Charnley category and preoperative EQ-5D (except from ∆EQ-5D). For the VAS scores, 0 indicated worst state of pain and satisfaction, whereas 100 indicated best possible state. Improvement in quality of life (∆EQ-5D) was estimated as the difference between preoperative and present EQ-5D index scores multiplied by 100.

In all analyses, p-values less than 0.05 were considered statistically significant. All tests were two-sided. The analyses were performed using PASW statistical software version 18.

The quality of radiological measurements was confirmed by Intraclass Correlation Coefficient (ICC), model ICC(3.1) and ICC(3.2) [22], measured for each individual angle.

## Ethics

The study was approved by the Regional Committee for Research Ethics in Western Norway (date of issue 2009/03/19, registration number 051.09) and the Norwegian Data Inspectorate (NSD) (date of issue 2009/05/15, registration number 21310).

## Results

Patients in the CON group were more often female (p < 0.05), more often operated with cemented prostheses (p < 0.01) and had a higher ASA score (p < 0.01). There was no difference in age, Charnley category and diagnosis (p > 0.05, Table 1).

**Table 1:** Patient characteristics of the groups

	**CON**	**CAOS**	**P-value**
Number of patients	95	103	
Mean age (min-max)(SD)	70.1 (49.0-89.5)(9.1)	68.7 (42.6-88.5)(9.2)	0.87
Sex (% female)	66.3%	50.5%	0.02
Pre-operative EQ-5D index score (SD)	49.3 (19.4)	45.1 (22.3)	0.21
ASA score			
1	10	9	<0.01
2	53	83	
3	19	9	
Unknown	13	2	
Type of prosthesis			
Profix cemented	58	26	<0.01
Profix uncemented	4	63	
Profix reversed hybrid	0	1	
Profix hybrid	33	13	
Operation time (min-max)(SD)	101.2 (57–250)(23.6)	90 (53–140)(17.4)	<0.01
Charnley category			
A	19	33	0.57
B	10	16	
C	35	42	
Unknown	31	12	
Diagnosis			
Osteoarthritis	90	98	0.98
Rheumatoid arthritis	1	1	
Other	4	4	

By 2011/12/31, six of the prostheses had been revised after the primary operation, three in each group. In the CON group, there were two revisions because of infection and one because of pain and poor function. In the CAOS group, two prostheses were revised due to infection and one because of instability.

### Radiographs

#### Coronal plane alignment

**Figure 3:**
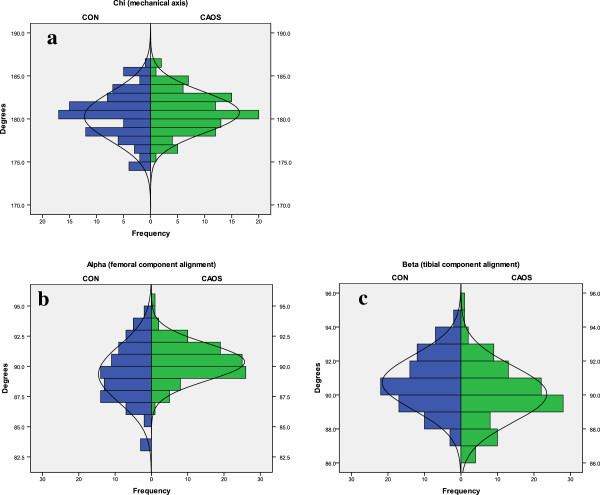
**Frontal plane alignment.** Values less than 180° for chi angle and 90° for alpha or beta represent valgus. An outlier is defined as more than ±3° from ideal angle measurement. 3**a**: *Chi* (mechanical axis). Outliers are 20% for CAOS and 25% for CON (p = 0.37). 3**b**: *Alpha* (femoral component alignment). Outliers are 5% for CAOS and 18% for CON (p < 0.01). 3**c**: *Beta* (tibial component alignment). Outliers are 8% for both CAOS and CON (p = 0.58).

For the *chi* angle (Figure 3a), 80% of the knees in the CAOS group were within ±3° of the ideal, compared to 75% in the CON group. The difference was not statistically significant (p = 0.37). Mean measurement (Table 2) was 180.3° in the CON group and 180.7° in the CAOS group. The difference was not statistically different (p = 0.23). Mean measurements of individual femoral and tibial component (*alpha* and *beta*, respectively) differed statistically in the two groups, but all mean measurements were within ±1° of expected ideal (Table 2).

**Table 2:** Angle measurements in CON and CAOS and inter- and intraclass correlation coefficients

**Angle**	**CON**	**CAOS**	**P-value**	**Inter-class correlation coefficient**	**Intra-class correlation coefficient**
Chi (min-max)(SD)	180.3 (174–186)(2.83)	180.7 (175–187)(2.38)	0.23	0.90	0.83
Alpha (min-max)(SD)	89.4 (84–95)(2.38)	90.3 (87–95)(1.52)	<0.01	0.92	0.89
Beta (min-max)(SD)	90.7 (87–94)(1.61)	90.0 (87–95)(1.66)	<0.01	0.95	0.91
Gamma (min-max)(SD)	4.39 (0–11)(2.39)	7.22 (0–16)(3.51)		0.95	0.81
Sigma (min-max)(SD)	89.9 (84–95)(2.26)	86.2 (79–95)(2.96)		0.98	0.95

For gamma and sigma, target value was different for CON and CAOS; consequently p-value is not shown.

ICC is >0.80 for all angle measurements, which is considered a good reliability.

With conventional technique, 18% of the femoral components (*alpha* angle) were outside 3° of ideal, versus 5% in the navigated group (Figure 3b), and the difference was statistically significant (p < 0.01). For the tibial component (*beta* angle), the number of outliers was 8.4% in the CON group and 7.8% in the CAOS group (Figure 3c), which was not a statistically significant difference (p = 0.58).

#### Sagittal plane alignment

**Figure 4:**
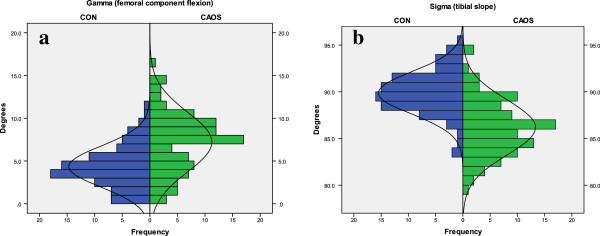
**Sagittal plane alignment.** In the CON group, ideal angles are 0-10° for *gamma* and 86° for *sigma*. In the CAOS group, the surgeon has adjusted the alignment to the patient’s anatomy. Thus, the angles had a wider range compared to the conventional group. 4**a**: *Gamma* (femoral component flexion). Large angles indicate high degree of femoral component flexion. 4**b**: *Sigma* (slope of tibial component). Angles less than 90 indicate posterior slope.

The *gamma* angle expressed the femoral flexion-extension. In the CON group, mean measurement was 4.4° and range 0-11°. In the CAOS group, mean and range was 7.2° and 0-16°, respectively (Figure 4a). The tibial slope (*sigma*) had a mean of 90 degrees and a range from 84 to 95 degrees in the conventional group. In the navigated group, mean tibial slope was 86 degrees, and the range was 79 to 95 degrees (Figure 4b).

Twenty randomly chosen patients (ten from each group) were measured twice by the observer and also by a second independent observer (ØG), to find the intra- and interobserver variabilities. The quality of measurements was confirmed by intraclass correlation coefficients (ICC). ICC was more than 0.8 for all angle measurements, which is considered a good reliability (Table 2).

### Questionnaire

We received questionnaires from 164 (83%) patients. The response rate was 74% in the CON group and 91% in the CAOS group. Total response rate for females was 83% and for males 83%. Median time from operation to completing the questionnaire was 3.3 (2.1-4.2) years in the CON group and 2.2 (1.5-3.7) years in the CAOS group.

**Figure 5:**
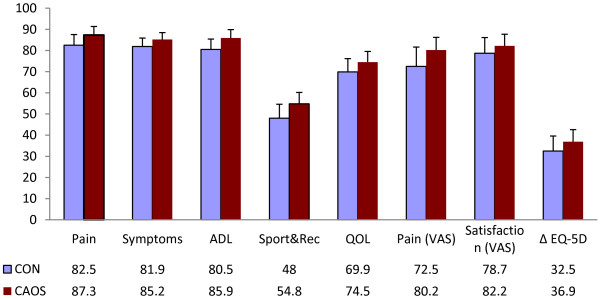
**Questionnaire outcomes.** Mean outcome scores for CON and CAOS. The first 5 outcomes represent the KOOS subscales. Results are adjusted for age, sex, fixation, Charnley category and preoperative EQ-5D index score (except for ΔEQ-5D). Outcomes were measured on a scale from 0 (worst) to 100 (best).

In the unadjusted analysis, we observed no differences between the CON group and the CAOS group for the KOOS sub-scales pain, symptoms, ADL and QOL, with all p-values >0.2. In the sub-scale Sport and rec, the CON group scored 46.4 and the CAOS group scored 55.8 (p = 0.03) In the adjusted analysis, there were no statistical difference in any of the KOOS sub-scales, but there was a trend towards higher score in all sub-scales for patients in the CAOS group (Table 3, Figure 5). Mean KOOS ADL score was 84 in the CON group and 86 in the CAOS group at two years. This coincides with the reference data for KOOS ADL; in the age group 55–74 it is 86 for men and 77 for women. In the age group 75–84 years, it is 76 for men and 83 for women [23]. Patients in the CAOS group also had a higher score in VAS for pain and satisfaction and ∆EQ-5D, but the differences were not statistically significant (all p-values >0.2) (Table 3, Figure 5).

**Table 3:** Mean difference in outcome between CON and CAOS

**Results KOOS**	**Diff**^**1,2**^	**(95% CI)**	**P-value**
Pain (SD)	-4.8	(-11.7, 2.0)	0.2
Symptoms (SD)	-3.4	(-8.8, 2.1)	0.3
ADL (SD)	-5.4	(-12.1, 1.3)	0.2
Sport and rec (SD)	-6.8	(-15.9, 2.2)	0.1
QOL (SD)	-4.6	(-13.1, 4.0)	0.3
Pain (VAS)	-7.7	(-19.4, 4.1)	0.2
Satisfaction (VAS)	-3.6	(-13.4, 6.3)	0.5
∆EQ-5D	-4.4	(-13.9, 5.1)	0.4

^1^Differences = mean scores among CON minus mean scores among CAOS. Negative values are in favor of CAOS.

^2^Differences in mean outcomes are adjusted for age, sex, fixation, Charnley category and preoperative EQ-5D index score (except for ∆EQ-5D).

**Figure 6:**
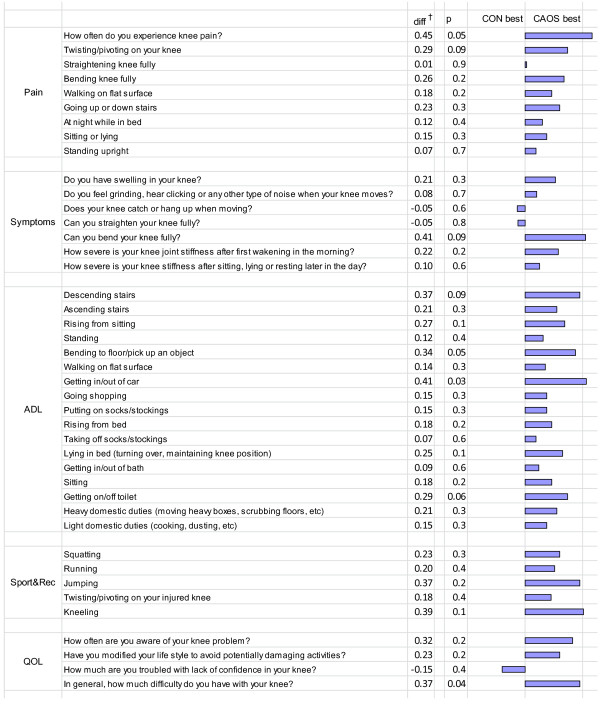
**Mean differences in outcome (detailed questions from KOOS) between CON and CAOS.** *Difference is equal to mean score among CON and CAOS (positive values are in favor of CAOS), Adjusted for age, gender, diagnosis, fixation method, Charnley category and preoperative EQ-5D index scores in a multiple linear regression model. With a Bonferroni correction, the significance level is set at p < 0.001. Consequently, none of the single questions in KOOS are statistically significantly in the groups. KOOS = the Knee Injury and Osteoarthritis Outcome Score; CON = conventional technique; CAOS = computer assisted orthopedic surgery; ADL = function in daily living; Sport/rec = function in sport and recreation; QOL = knee related quality of life.

In the analyses of the detailed questions from KOOS (Figure 6), there was also a trend towards better results for CAOS. We observed a clinically significant difference in three questions, considering how often the patient experienced knee pain (p = 0.05), ability to bend the knee fully (p = 0.09) and difficulties in getting in/out of car (p = 0.03). The observed differences were all in favor of CAOS.

The possible difference for inliers and outliers for the *sigma* and *gamma* angle were investigated in the three situations; "Can you bend your knee fully?", "Getting in/out of car?" and "Getting on/off toilet?" in the CAOS group of 103 knees. The analyses were adjusted for the same variables as before. We found no statistical significant differences except for the question "Can you bend your knee fully?" where we found p-value = 0.044. Internally validation of the statistical model by use of bootstrapping (p = 0.08) could however not confirm this finding.

### Operation time

The operation time was 101 minutes in the CON group and 90 minutes in the CAOS group (Table 4). The difference was statistically significant (p < 0.01). By exclusion of uncemented prostheses in both groups, there was no longer any statistically significant difference (101 min for CON, 97 min for CAOS; p = 0.37).

**Table 4:** Operation time

**Operation time**	**CON**	**CAOS**	**P-value**
All prostheses (min-max)(SD)	101.2 (57–250)(23.6)	90 (53–140)(17.4)	<0.01
Cemented prostheses only (min-max)(SD)	101.1 (65–250)(28.0)	96.8 (65–123)(16.0)	0.37

CON = conventional technique; CAOS = computer assisted orthopedic surgery.

## Discussion

We compared the outcome of computer navigation versus conventional method in total knee arthroplasty by one single surgeon. According to our results, CAOS can reduce the number of outliers for the femoral component in coronal plane alignment. Measurements for mechanical axis and tibial component did not differ statistically significantly. In the questionnaires, we observed that CAOS reached a higher score in all subscales, but the differences were not statistically significant. Number of revisions and operation time did not differ in the two groups.

### Radiographs

On radiographs, the average measured angles differed significantly in all angles except from the mechanical axis, but all mean measurements in the frontal plane were within ±1° of expected ideal. For the femoral component, there were statistically significant fewer outliers in the CAOS group compared to the CON group. For HKA alignment and alignment of the tibial component, there were also fewer outliers in the navigated group, but the difference was not statistically significant. Previous studies have reported that patients operated with conventional technique have a higher proportion of outliers compared to TKA operated using computer navigation [2,8,24].

On sagittal radiographs, the range of measurements was wider in CAOS compared to CON. When operating by conventional method, the intramedullary rods determine the tibial slope and femoral flexion. In contrast, the navigation system allows the surgeon to modify the femoral flexion and tibial slope, according to the patient’s original anatomy. In our study, the surgeon aimed for more flexion of the femoral component and a more posterior tibial slope in the CAOS group. This was thought to improve flexion and with that also function scores [25,26]. In the CON group, mean measurement was 90° for tibial slope, which is 4° more than ideal of 86°.

### Questionnaire

There was no statistically significant difference in VAS score, ∆EQ-5D or any of the KOOS main categories two years after surgery. However, we found a clinically significant difference in three single questions in the KOOS score, all in favor of CAOS. Furthermore, CAOS had a better outcome in 39 of 42 questions, but the findings were not statistically significant and thus of uncertain importance. Two randomized controlled trials have previously found no clinical difference between CAOS and CON in scores of function and quality of life [27,28]. A prospective randomized trial and a recent follow-up study reported a higher Knee Society Score and Short-Form 12 physical scores for patients with coronal alignment within 3° of neutral, regardless of surgical technique [24].

### Operation time

Including all procedures, the CAOS group had an average operation time of 11 min shorter than in the CON group. However, there was a considerable amount of uncemented prostheses in the CAOS group. By excluding all uncemented prostheses, the difference in operation time was 4 min in favor of CAOS, and the result was not statistically significant. Previous studies have reported longer operation time when using navigation [2,6,29]. However, other studies have found that surgery duration is reduced remarkably once the surgeon is experienced with navigation, and that the operation could be performed equally fast, or even quicker with CAOS when the surgeon is well-experienced [7,8]. A short-term register study on data from NAR [6], found that mean operation time in all Norwegian hospitals was 92 min with conventional total knee replacement and 107 min with navigation.

### Strengths and limitations

The strength of this study is that all the patients were operated by the same surgeon, and this surgeon was already experienced in both methods at the beginning of the study. The evaluation of the results represents a centre of high volume of knee replacement, which is considered most cost-effective [30]. On the other hand, we cannot tell from this study the outcome of an average surgeon or how many procedures needs to be done to achieve enough experience.

Radiological parameters were measured by an independent observer. Using conventional radiographs instead of CT postoperative, we were not able to compare rotation of the components. CT measurements are also considered more accurate [31]. Considering femoral flexion and tibial slope, the surgeon has made individual adjustments in the CAOS group, while this was not possible in the CON group. We do not have data for target value in each individual patient in CAOS, and cannot test deviation from aimed angle in these patients. Consequently, it is difficult to compare the groups to an expected ideal angle in the sagittal plane.

This study is retrospective, and the results are less conclusive than results from randomized clinical trials. The inclusion period is different in the two groups, and we cannot ignore the fact that there may have been an unintentional selection bias. A reasonable part of the prostheses in the CAOS group were uncemented. This affected the operation time in favor of CAOS, but we do not know whether it affected the placement of the components. The patients in the CON group are older, more often female and have a higher ASA score compared to the CAOS group. To reduce this difference, we have made adjustments for possible confounders when calculating the KOOS score using multiple linear regression analyses*.* Except from the preoperative EQ-5D, all questions were based on the patient’s experience during the previous week, and we consider the risk of recall bias as negligible.

### Future research

Several studies have been published on alignment in computer navigation, some of them with CT measurements, but non with RSA, which should be done. However, there is little information on how computer navigation affects the results at long term. Register studies and randomized studies with long term follow-up are required to explore the risk of revision and the outcome of loosening, pain and instability.

## Conclusions

Based on our results, the use of computer navigation in TKA slightly reduces the number of outliers in coronal alignment measurements of the femoral component. In the hands of an experienced surgeon, it is possible to perform the procedure in the same time schedule as with conventional technique. Navigation also makes it possible to adjust component placement to the patient’s anatomy. In an average patient population, there is no difference in functional outcomes and quality of life or in main categories of function scores, and the all-over clinical effects of CAOS are uncertain. Still, we observed that CAOS had a non-significant trend towards better outcome in all categories two years post-operatively. Short term results of revision were not affected.

## Abbreviations

CAOS: Computer assisted orthopedic surgery; CON: Conventional technique; TKA: Total knee arthroplasty; KOOS: Knee and osteoarthritis outcome score; VAS: Visual analogue scale; ASA: American society of anesthesiologists; NAR: Norwegian arthroplasty register; HKA: Hip-knee-ankle; ICC: Intraclass correlation; NDI: Norwegian data inspectorate; ADL: Function in daily living; QOL: Quality of life; RSA: Radiosterometic analysis.

## Competing interests

The authors declare that they have no competing interests.

## Authors’ contributions

GSD carried out the radiological measurements and drafted the manuscript. ØG did the radiological control measurements and participated in the development of the Radiological Measures Protocol. AMF and SHLL performed the statistical analyses. TJ included the patients, performed all TKA procedures and participated in the design of the study. SH participated in the development of the Radiological Measures Protocol. TES created the database and gave IT-support whenever needed. OF was the leader of the study, participated in its design and coordination and helped in drafting the manuscript. All authors read and approved the final manuscript.

## Pre-publication history

The pre-publication history for this paper can be accessed here:

http://www.biomedcentral.com/1471-2474/14/321/prepub
